# Do Norwegian Sami and non-indigenous individuals understand questions about mental health similarly? A SAMINOR 2 study

**DOI:** 10.1080/22423982.2018.1481325

**Published:** 2018-06-05

**Authors:** Tore Sørlie, Ketil Lenert Hansen, Oddgeir Friborg

**Affiliations:** a Department of Clinical Medicine, Faculty of Health Sciences, UiT The Artic University of Norway, Tromsø, Norway; b Department of Mental Health and Addictions, University Hospital of North Norway, Tromsø, Norway; c Centre for Child and Youth Mental Health and Child Welfare, RKBU North, Faculty of Health Sciences, UiT The Arctic University of Norway, Tromsø, Norway; d Department of Psychology, Faculty of Health Sciences, UiT The Arctic University of Norway, Tromsø, Norway

**Keywords:** Cross-cultural validation, Hopkins Symptom Checklist (HSCL-10), invariance analyses, Norway, patient-reported outcome measures (PROs), SAMINOR 2 questionnaire study

## Abstract

The Western culturally developed Hopkins Symptom Checklist (HSCL-10) is a self-report measure of mental distress widely used for both clinical and epidemiological purposes – also in the multiethnic epidemiological SAMINOR studies in Northern Norway, but without any proper cross-cultural validation. Our objective was to test invariance of the HSCL-10 measurements among Sami and the non-indigenous majority population in Northern Norway (participants in the SAMINOR 2 study) and whether the previously used HSCL-10 cut-off level (1.85) fits the Sami subgroups in the study. Participants belonged to Sami core, Sami affiliation, Sami background or majority Norwegian groups. The confirmatory factor analysis framework adapted for testing of measurement invariance showed no significant measurement invariance between the groups indicating that the HSCL-10 response scale predominantly was used in the same way and that significantly different meanings were not ascribed to the same set of questions. The cut-off criteria of 1.85 as indicative of psychological distress based on Norwegian data equal a score of 1.89, 1.94 and 1.91 in the Sami core, Sami affiliation and Sami background groups, respectively. Thus, the same cut-off criterion 1.85 may be safely used in all groups. However, one should still be looking for culture-specific expressions of mental stress.

## Background

Culture influences concepts of normality and pathology as well as the experience, expression, meaning and communication of symptoms [1]. This may have implications for identification, prevention and prognosis of mental disorders. Participants in the present study belonged to the Sami and the non-indigenous majority population in Northern Norway. Historically, the Sami were nomadic reindeer herders or small-scale farmers and fishermen along the coastline. Today, less than 10% are engaged in reindeer husbandry, and most live well integrated into the Norwegian majority population [2]. Compared with majority Norwegians, Sami tradition places greater emphasis on bringing children up to independence, robustness and tolerance for discomfort [3,4] and not having them to talk about mental problems [5]. Mental health stigma is stronger, and the Sami are more frequently using traditional treatment forms [6]. Discrimination and childhood violence are more frequently reported with their negative effects upon mental distress [7–9]. A rigorous Norwegian assimilation policy, which included use of boarding schools where the Sami language was forbidden, took place particularly between 1860 and 1960. However, with centuries of extended coexistence and cultural interaction between Sami and Norwegian settlements in the same geographical areas, most Sami today consider themselves as having both a Norwegian and a Sami identity [2]. Besides, the majority of contemporary Sami, except perhaps those living in so-called “core Sami areas”, also have Norwegian as their mother tongue language. They therefore speak Norwegian language about as fluently as the majority population do, and some Sami children learn to speak both languages in kindergarten. However, the Sami still have a somewhat lower level of education, employment rate, income and living expectancy than majority Norwegians [10].

Health and living conditions among Sami and non-Sami groups in northern Norway have been compared in the epidemiological SAMINOR 1 and 2 studies [11,12]. Psychological distress was assessed with the patient-reported outcome measure (PRO) Hopkins Symptom Checklist (HSCL-10) [13]. In the SAMINOR 1 study, Sami males reported greater levels of psychological distress than ethnic Norwegians (7.3 vs 5.9%). In females, there were no differences (10.0 vs 10.2) [7]. In the SAMINOR 2 study, psychological distress was significantly higher in both Sami women (15.8 vs 13.0%) and men (11.4 vs 8.0%). Also posttraumatic stress symptoms were higher in the Sami: in women 16.2 versus 12.4 and 12.2 versus 9.1 in men [9]. However, these differences were small to negligible after adjustment for education, income, discrimination and resilience [8]. Similar results appeared in a meta-analysis of 19 studies comparing depression and anxiety among culturally diverse indigenous and non-indigenous populations in the Americas. Mainly based on clinical interviews, no differences in the 12-month prevalence rates of mental distress appeared. However, indigenous groups were at higher risk of PTSD [14]. Social origins of mental health problems in aboriginal peoples relate to cultural discontinuity and oppression [15]. However, resilience factors such as individual coping capacity as well as unity and support within family and society can protect against distress [8].

Although some studies report adequate cross-cultural equivalence of many assessment methods used [16,17], others show that reported prevalence rates of common mental disorders across countries strongly depend on the inclusion of culture-specific expressions of mental distress. This was clearly illustrated in a study comparing prevalence rates of mental disorders in 3 population-based surveys drawn from the Mekong Delta region of Vietnam, Vietnamese immigrants residing in New South Wales in Australia, and an Australian-born population. Based on the Composite International Diagnostic Interview (CIDI) assessing Western-defined mental disorders, the prevalence rate of mental disorders for Mekong Delta Vietnamese was 1.8%, 6.1% for Australian Vietnamese and 16.7% for Australians. In addition, the Vietnamese surveys also applied the Phan Vietnamese Psychiatric Scale (PVPS) designed to identify culturally relevant idioms and expressions of psychological distress in the Vietnamese ethnic group. Inclusion of PVPS mental disorders increased the prevalence rates to 8.8% in the Mekong survey and to 11.7% for the Australian Vietnamese [18]. Another rating-scale adapted to indigenous people is The Kessler Psychological Distress Scale (K10). It appears to be psychometrically sound for use as a broad measure of non-specific psychological distress for First Nations people living in Canada [19]. No such concepts or scale have been developed or tested for the Sami population.

Other transcultural epidemiologic studies, such as a WHO study [20] reporting considerable variation of mental disorders across countries, have typically used Western culturally developed psychiatric diagnostic criteria exclusively [21].

The constructs that such scales intend to capture may be defined qualitatively by triangulating patient experiences, expert opinions and literature reviews [22]. Typically, such concepts are operationalised through a list of multiple questionnaire items, with the intention behind each item to represent an aspect of the targeted mental health concept [23]. Responses to items supposed to represent a single shared attribute are usually summed in a composite measure. Because of the non-observable nature of such measurements, they are often referred to as latent variables [24,25]. If those being compared ascribe different meanings to the same set of questionnaire items, epidemiological studies linking questionnaire data with health outcomes thus risk concluding erroneously about prevalence rates of the targeted health issues as well as corresponding risk or protective factors in comparisons of ethnic groups.

The use of PROs, such as measures of mental distress or quality of life, thus presupposes a proper cross-cultural validation before using it comparatively in epidemiological mental health research.

The overall quality of health measurement scales depends on its reliability and validity. Reliability refers to the precision or the accuracy of the summed scores given by the scale, typically as internal consistency coefficients (e.g. Cronbach’s alpha or Raykov’s rho) or test-retest correlations indicating stability. Construct validity is a broader evaluation of whether the scale fits with a theoretical system, for example by producing correlations as expected with theoretically related constructs. Discriminative validity evaluates to what extent a scale captures (or measures) particular hypothesised facets of a construct (e.g. to what extent the subscales measure distinct aspects of the construct). This may be examined with confirmatory factor analytic (CFA) approaches [24]. An advantage of CFA is the possibility for between group comparisons of all aspects of a measurement model (e.g. factor loadings or factor means), thus enabling cross-cultural comparisons.

PROs may not replace a structured clinical diagnostic evaluation of an individual, which is the gold standard in psychiatric examinations [26]. Nevertheless, cut-off scores are very useful proxy indicators of psychological distress for use in comparisons of the health status in different strata of a population. Hence, a large number of studies have searched for the most optimal cut-off scores that separate true pathology from true normality.

When the first SAMINOR study was planned about year 2000, The Hopkins Symptom Checklist (HSCL-10) [27] was chosen as a PRO measure of psychological distress. About the same time, it had been used in the Survey of Level of Living in Norway in 1998 [28] and to measure psychological stress among immigrants in the Oslo Health Study [29]. Although originally developed to measure the efficacy of psychotropic drugs in adult North American outpatients, it has been used in studies of both youth and adults in the general population worldwide. The cross-ethnic equivalence of the HSCL-21 has proven satisfactory in European American, African American and Latino College students [16]. A good concordance between HSCL-10 and a Pakistani indigenous instrument measuring mental distress has been demonstrated [17]. However, its validity among the Sami people in Norway has previously not been tested. Hence, the cut-off level of 1.85 for the HSCL-10 found in the Norwegian majority population [13] has been used in the SAMINOR 1 and 2 studies [11,12].

A suitable method for examining whether the measured construct (i.e. HSCL) has the same psychometric properties across ethnic groups is the confirmatory factor analysis framework adapted for testing of measurement invariance [24]. Support of measurement invariance suggests that individuals from different ethnic groups interpret the items and use the response scale comparably, thus, ascribing similar meanings to the same set of questions. Tests of invariance may be examined at several levels, such as factor model, factor variances/covariances, or item loadings, intercepts or errors. The present study focused on testing the invariance at an item level exclusively and set free those measurement model parameters that worked differently between the ethnic groups, in order to produce adjusted estimated item raw scores equated for the same underlying latent trait score.

The aims of this study are to test invariance of the HSCL-10 symptom measure among Sami and the non-indigenous majority population in Northern Norway (participants in the SAMINOR 2 study), in order to decide whether the previously used HSCL-10 cut-off level (1.85) also fits the Sami subgroups.

## Material and methods

### The SAMINOR 2 study

This second survey of the population-based study on health and living conditions in regions with Sami and Norwegian populations – the SAMINOR 2 questionnaire study – is a cross-sectional epidemiological study. The SAMINOR study was conducted by the Centre for Sami Health Research, Department of Community Medicine, UiT – the Arctic University of Norway. The study is thoroughly described with regard to the target population, study variables and data collection procedures in a paper by Brustad et al. [12].

### Sample

All residents aged 18–69 years were invited by mail (N = 44,669). With 1,424 invitations returned unopened, 43,245 persons were eligible. Among these, 11,600 persons consented by returning the questionnaire (27% participation rate). Subjects also dropped out of the analyses due to completely missing data about ethnicity (95 cases), discrimination (515 subjects), resilience (181 subjects) and background information (744 subjects, lacking covariate information), leaving 10,065 subjects available for analysis.

### Stratification on ethnicity

The statistical analyses were stratified on ethnicity, hence presenting descriptive and inferential statistics separately for the identified ethnic subgroups. Three types of questions were used to decide the ethnicity of the participants: 1) language spoken at home (Norwegian, Sami, Kven or other language either by the person, the parents or the grandparents), 2) ethnic self-identification either as Norwegian, Sami, Kven or Other, and 3) ethnic background either as Norwegian, Sami, Kven or Other. Based on these questions, the following 5 ethnic subgroups were created: 1) “Norwegian” if only Norwegian markers were endorsed (*N *= 5,608), 2) “Norwegian KO” if a *Kven* or an *Other* ethnicity marker were additionally endorsed, hence representing a mixed ethnic category (*N *= 1,969: among these *n *= 1,389 endorsed at least 1 *Other* marker, while *n *= 700 endorsed at least 1 *Kven* marker, and *n *= 120 endorsed a combination of *Other* and *Kven* markers), 3) “Sami background” (*N *= 1,097) if identifying oneself as Norwegian but additionally reports Sami ancestry (parents/grandparents speaking Sami, or having parents with a Sami background), 4) “Sami affiliation” (*N *= 1,459) if reporting 1 or 2 Sami markers (the person speaks Sami, self-identify as Sami, or reports Sami ethnic background), and 5) “Strong Sami” (*N *= 1,372) if a subject endorsed all 3 Sami markers.

### The Hopkins Symptom Checklist-10 (HSCL-10)

The HSCL is a 10-item short version of the 90-item Symptom Check List (SCL-90). It is a PRO that rates the presence of symptoms during the last 4 weeks related to depression (6 items) and anxiety (4 items): sudden fear, frightened/anxious, faintness/dizziness, tense/upset, blame yourself, insomnia/sleeplessness, dejected/sad, feeling useless, everything is a struggle and hopelessness. It uses a 4-point scale (from 1 – not at all to 4 – very much) with higher mean scores indicating more mental distress (range 1–4).

### Ethics

The Norwegian Data Protection Authority (Datatilsynet) approved the data collection and storage. Written informed consent was obtained form all participants. The Regional Committee for Medical and Health Research Ethics of Northern Norway (REK-Nord) and SSB approved the study.

## Psychometric analyses

### Evaluation of measurement model fit

Mplus version 7.11 by Muthén and Muthén [30] was used for estimating the confirmatory factor analysis (CFA) models. The HSCL item scores were heavily positively skewed (range Z skewness = 62.6–181.8, *M* = 98.0), and kurtotic (range Z kurtosis = 32.0–437.5, *M* = 126.0), which is normal for low prevalent phenomena. Hence, the robust maximum likelihood (MLR) estimator was used. The fit of the factor model was evaluated as the degree of model discrepancy (RMSEA-root mean square error of approximation) and degree of relative fit (CFI-comparative fit index; and NNFI-non normed fit index). RMSEA values below < .06 are preferable [31], while values for CFI/NNFI should minimally pass > .90 [32] or preferably > .95 [33]. Absolute model fit (i.e. chi-square) is also reported but not interpreted as minor and ignorable model misspecifications will be flagged as significant in large sample sizes [34], as in the current study.

### Different forms of measurement invariance

The most basic is *form invariance*, which simply requires the same measurement model (i.e. same number of factors and factor loading patterns) to fit the data reasonably well in all groups. This is a necessary assumption, but in order to understand whether subjects from different ethnic strata treat the HSCL item scores differently, one needs to examine group differences on an item level: *metric, error* and *scalar* invariance. *Metric invariance* is the most important as it indicates whether individuals interpret the items similarly by examining if the factor loadings are equal across the groups. Equal loadings mean that a change in item unit raw scores reflects a comparable change in the underlying latent trait score in all groups, thus measuring the construct similarly in all groups. Items with higher loadings are also more sensitive construct indicators. The 2 remaining, *error* and *scalar*, are more related to reliability and scaling issues rather than the construct per se. They are more frequently violated and less important than the former, but important to adjust for statistically. *Error invariance* examines whether the item reliabilities are equal, thus indicating whether the items measure the latent construct with comparable precision in all groups. Variance in the error parameters are generally prevalent and not necessarily problematic as the items may still measure the construct reliably, albeit with a different degree of precision in the groups. *Scalar invariance* examines if all ethnic subgroups use the range of the response scale differently, thus examining the placement of the latent intercept for a particular item (where it crosses the y-axis given a latent factor mean score of 0). The latent intercept is in practice quite close to the observed mean score. If the latent intercepts are systematically higher in certain ethnic groups for some items, this would indicate that these subgroups endorse endpoint scores (e.g. extremely high) more often compared to other groups. Hence, the raw composite (total) score would be higher in those groups despite the groups are equal on the latent trait score. The number of variant factor loadings should therefore be low, whereas variances in the 2 latter parameters (errors and intercepts) are more frequent and may be adjusted for.

### Evaluation of measurement invariance

A series of nested multi-group CFA analyses were conducted to determine HSCL items that were variant and invariant across the ethnic groups [35]. The first, or baseline model, estimated all parameters related to item precision (error variances), factor loadings, item intercepts (scaling position) and latent factor variances as equal. These restrictions were successively loosened until the improvement in model fit stopped, starting with the parameter having the largest modification index. A parameter was thus set free if the rescaled chi-square difference test was significant [36]. The error variance parameters were first specified as equal and successively loosened until the chi-square difference test no longer improved significantly. A similar process was run for the factor loadings, and for the latent item intercepts. All factor variances were finally loosened. This process ensured that any sample specific (or, ethnic group) model parameter differences were statistically adjusted for in the final measurement model. The adjusted measurement parameters were next used to estimate the raw score for each item using the following formula: HSCL *item*_k_ = *icept*_k_ + *factor score* × √*factor variance* × *loading*_k_. The intercepts and the loadings were variant for some of the items, as identified by the procedure described above. Estimating the measurement model with free parameters for those items that work significantly differently in certain ethnic subgroups will produce adjusted estimated item raw scores equated for the same underlying latent trait score. Separate factor variances for the ethnic groups were allowed. The factor score needed to produce an average estimated raw score of 1.85 (the HSCL cut-off score) could then be solved.

## Results

### HSCL-10 factor model fit

The HSCL one-factor model indicated tenable to mediocre fit if based on all 10 items as the RMSEA index ranged between .062 and .085 (see Table 1). Separating the model in 2 latent factors, one for depression and another for anxiety, was not tenable as the anxiety factor only accounted for 2 items of the 4 intended anxiety items. A one-factor model accounting for the 6 depression items exclusively fitted the data considerably better (RMSEA ranging between .045 and .059). These items are thus more valid indicators of depression compared to using all items. Since the fit of the 10 items were reasonable and this version is much used in epidemiological research, all items were included in the subsequent invariance analyses comparing the latent item properties across the ethnic groups.

**Table 1.** Invariance testing of the responses to the HSCL-10 symptoms questionnaire.

	Norw	Norw-Kven *M* diff (*t*)	Sami Core	Sami Affilitation	Sami Background
*Model fit HSCL-10 All 10 items*					
*χ*^2^(df)	950.4^a^ (35)	334.2^a^ (35)	213.4^a^ (35)	313.3^a^ (35)	306.8^a^ (35)
RMSEA/SRMR/TLI	.**069**/.043/.891	.**067**/.040/.912	.**062**/.039/.923	.**074**/.040/.908	.**085**/.045/.880
*Model fit HSCL-6 Depr items only*					
*χ*^2^(df)	140.3^a^ (9)	41.8^a^ (9)	49.6^a^ (9)	47.1^a^ (9)	36.5^a^ (9)
RMSEA/SRMR/TLI	.**053**/.022/.962	.**045**/.017/.978	.**059**/.027/.958	.**056**/.022/.966	.**055**/.021/.970
*Invariance testing of HSCL-10*					
**M0: all equal ^1^**		Unadj fit: χ^2^ = 9835.6^a^, *df* = 318, ***RMSEA* = .118**, *SRMR* = .284, *TLI* = .730			
		Adj fit: χ^2^ = 11,583.2^a^, *df* = 478, ***RMSEA* = .106**, *SRMR* = .200, *TLI* = .700			
Latent mean, unadj ^2^	0 (reference)	.057 (4.31^a^)	.055 (3.69^a^)	.098 (6.32^a^)	.068 (4.02^a^)
Latent mean, adj ^3^		.028 (2.16^c^)	.025 (1.66)	.074 (4.93^a^)	.042 (2.56^b^)
**M1: free residuals ^4^**		Unadj fit: χ^2^ = 2375.3^a^, *df* = 254, ***RMSEA* = .063**, *SRMR* = .062, *TLI* = .925			
		Adj fit: χ^2^ = 2921.5^a^, *df* = 414, ***RMSEA* = .054**, *SRMR* = .047, *TLI* = .922			
Latent mean, unadj ^2^	0 (reference)	.029 (4.16^a^)	.034 (4.28^a^)	.054 (6.39^a^)	.034 (3.77^a^)
Latent mean, adj ^3^		.013 (1.86)	.018 (2.20^c^)	.041 (5.00^a^)	.020 (2.25^c^)
**M2: free loadings ^5^**		Unadj fit: χ^2^ = 2338.7^a^, df = 242, **RMSEA = .064**, SRMR = .062, TLI = .922			
		Adj fit: χ^2^ = 2895.2^a^, df = 402, **RMSEA = .055**, SRMR = .047, TLI = .920			
Latent mean ^2^	0 (reference)	.031 (4.44^a^)	.037 (4.57^a^)	.056 (6.59^a^)	.036 (3.99^a^)
Latent mean, adj ^3^		.016 (2.23^c^)	.021 (2.59^b^)	.044 (5.29^a^)	.022 (2.54^c^)
**M3: free intercepts ^6^**		Unadj fit: χ^2^ = 2089.0^a^, df = 206, **RMSEA = .065**, SRMR = .061, TLI = .918			
		Adj fit: χ^2^ = 2715.5^a^, df = 366, **RMSEA = .056**, SRMR = .046, TLI = .917			
Latent mean ^2^	0 (reference)	.028 (3.23^a^)	.035 (3.59^a^)	.053 (5.23^a^)	.027 (2.55^c^)
Latent mean, adj ^3^		.013 (1.51)	.019 (1.93)	.039 (3.97^a^)	.017 (1.63)
**M4: free variances ^7^**		Unadj fit: χ^2^ = 2086.8^a^, df = 202, **RMSEA = .066**, SRMR = .046, TLI = .916			
		Adj fit: χ^2^ = 2710.5^a^, df = 362, **RMSEA = .056**, SRMR = .037, TLI = .916			
Latent mean ^2^	0 (reference)	.028 (3.22^a^)	.035 (3.58^a^)	.054 (5.21^a^)	.028 (2.54^c^)
Latent mean, adj ^3^		.014 (1.54)	.019 (1.94)	.040 (3.96^a^)	.018 (1.65)

^a^*p* < .001, ^b^*p* < .01, ^c^*p* < .05. ^1^ All loadings specified as 1, all intercepts and all residuals constrained equal across all items and all groups. ^2^ The zero latent mean score (*M *= 0) equals a HSCL sum score of 1.321 in the Norwegian sample. ^3^ HSCL item scores in addition adjusted for the covariates age, gender, income and education. ^4^ Nine error variances in addition set free (except item 8), loadings and intercepts also set free across items but constrained equal across groups. ^5^ Same as model M1, in addition 3 loadings set free across groups (item 4, 6 and 9). ^6^ Same as model M2, in addition 9 intercepts set free across groups (except for item 7 to achieve convergence). ^7^ Same as model M3, but factor variances in addition set free.

### Invariance in psychiatric symptoms (HSCL-10) across ethnic groups

The first model (M0), which was specified with the same measurement assumptions as that used in a conventional Student t-test (see note to Table 1), showed that the HSCL latent mean scores were significantly higher in the Sami groups compared to the Norwegian group, as previously reported. Since the education and income levels are lower in Sami compared with Norwegian subjects [10], the HSCL item scores were adjusted for these variables in addition to gender and age. The latent mean differences shrunk but retained significance. Four additional models were specified, which successively loosened the measurement constraints by freeing the latent errors, loadings, intercepts and factor variance parameters until model fit stopped improving significantly. In model M1, 9 errors were successively freed until the model improvement stopped (equal vs. free residuals; *SB* diff χ^2^ (36) = 203.1, *p* < .001). The ethnic group differences shrunk but retained significance. The average item errors ranged between 46.7% and 51.8% (SD 11.1–12.7%) across the subgroups. These differences were in practice small despite the significant parameter differences. In Model M2, 3 factor loadings were freed until improvement in model fit stopped (*SB* diff χ^2^ (12) = 21.70, *p* < .04). The average loadings ranged between .656 and .693 (*SD* .077–.098); hence, these differences were also small in magnitude. In Model M3, 9 intercepts were freed (*SB* diff χ^2^ (36) = 118.5, *p* < .001). The average item intercepts were 1.319 in the Norwegian group and ranged from 1.311 (Sami core) to 1.327 (Sami background) in the other groups. Despite Norwegians systematically endorsing higher endpoint scores than the others, these differences were also small in practice. In Model M4, all factor variances were additionally freed (*SB* diff χ^2^ (4) = 17.2, *p* = .002). The latent mean group differences ceased to be significant in Model M3 and M4, except for the “Sami Affiliation” group, which still had slightly higher latent scores following the adjustment for the different psychometric properties across the ethnic groups.

The item graphs portray the relationship between raw item scores and latent HSCL trait scores. These graphs show that the Sami groups slightly exaggerate their symptom reporting compared to majority Norwegians, which however disappear after adjusting for socioeconomic covariates and ethnic group differences in the measurement model properties.

The HSCL raw scores as estimated from the adjusted measurement parameters are presented in Table 2. These scores were based on a latent factor score of 1.315 in order to produce an average HSCL score of 1.85 in the majority Norwegian group, which represented 8.5% of the distribution (above this cut-off). Higher estimated raw scores imply 2 things: a higher prevalence of these symptoms as they are endorsed more often, but also less sensitivity as indicators of underlying mental health problems. Hence, the 3 first HSCL items (sudden fears, frightened and faintness, having low scores) are less prevalent symptoms but more sensitive indicators of mental distress than the remaining HSCL items (see Table 2). The 2 items with the highest estimated raw scores (insomnia and everything is a struggle) are the most prevalent symptoms but the least sensitive indicators. The differences in the estimated item scores between the ethnic groups were in general minor, except for item 6 (insomnia/sleeplessness). This item is thus less prevalent and correspondingly more sensitive of mental distress in the Sami core group exclusively. Finally, if the adjusted measurement model parameters are used to compare the overall average HSCL raw score, the cut-off criterion of 1.85 in majority Norwegians would equal a cut-off score of 1.89, 1.94 and 1.92 in the Sami core, Sami affiliation and Sami background groups, respectively.

**Table 2.** Estimated HSCL item scores using the adjusted measurement model parameters.

	Estimated raw score (range 1–4)					
HSCL item	Norw	Norw-Kven	Sami core	Sami affiliation	Sami backgr	*diff*
Sudden fears	1.40	1.44	1.46	1.46	1.44	.06
Frightened or anxious	1.65	1.71	1.75	1.74	1.69	.10
Faintness or dizziness	1.70	1.77	1.72	1.78	1.76	.08
Tense or upset	1.97	2.02	2.04	2.06	2.02	.09
Blame yourself	1.99	2.05	2.03	2.10	2.02	.11
Insomnia/sleeplessness	2.06	2.20	1.86	2.14	2.18	.34
Dejected or melancholic	1.94	2.02	2.02	2.05	2.00	.11
Useless, or little value	1.85	1.90	1.90	1.94	1.90	.09
Everything is a struggle	2.06	2.15	2.14	2.14	2.14	.09
Hopeless about future	1.88	1.95	1.99	1.96	1.96	.11
*M*	1.850	1.921	1.891	1.937	1.911	.087

Diff = Largest difference.

## Discussion

The HSCL-10 is a widely used symptom inventory aimed at detecting or screening for depression and anxiety. The symptom descriptors may however be experienced differently across cultures and consequently arouse quite different interpretations or meanings. The use of a PRO, such as the HSCL-10, thus presupposes a proper cross-cultural validation before using it for comparative purposes in epidemiological research on mental health.

We used a confirmatory factor analysis (CFA) framework to examine whether respondents from the majority Norwegian culture interpret the HSCL items similarly as respondents from Sami populations in Norway. The CFA methodology supposes that all measurement model parameters, for example, factor loadings, factor variances, latent intercepts and errors, are equal across the groups, or invariant. Since the CFA modelling approach is highly flexible, these strict requirements may be loosened by allowing separate parameters to be estimated within each group, thus fitting a measurement model that fit the particular ethnic group best. This is considered a suitable method for examining whether the measured constructs reflect true group differences that are not contingent on group-specific features unrelated to the constructs of interest [24,37].

The degree of measurement variance in our study was in general low, thus suggesting that individuals from the different ethnic groups in the present study interpret the HSCL-10 item descriptors and their response scale quite equal. Hence, they do not ascribe significantly different meanings to the same set of questions.

This finding indicates that most Sami adopt the Western Norwegian mental health concept as measured by the HSCL-10. This may relate to fact that most Sami today consider themselves as having both a Norwegian and Sami identity [2]. They also live their lives well integrated in the Norwegian society, and most speak Norwegian language about as fluently as the majority population do. In fact, some Sami children today learn to speak both languages in kindergarten, thus being bilingual. The minor differences in the understanding of the item descriptors may also relate to centuries of extended coexistence and cultural interaction between Sami and Norwegian settlements in the same geographical areas. The Norwegian assimilation policy period, during which the Sami language was prohibited in schools between 1860 and 1959, may also have contributed.

An implication is that the often-used cut-off criteria of 1.85 as indicative of psychological distress based on Norwegian data equal a score of 1.89, 1.94 and 1.91 in the Sami core, Sami affiliation and Sami background groups, respectively. As the exaggeration of symptom reporting in the Sami groups is minor in relation to the Norwegian group, the same cut-off criterion may be safely used in both majority Norwegian and Sami populations.

The differences in the estimated item raw scores between the ethnic groups were in general minor, except for item 6 (insomnia/sleeplessness), which appears a more sensitive indicator of mental distress in the Sami core group exclusively. It is well known that disturbances in emotion regulation and sleep are common aspects of anxiety and depression and that changing quality in sleep is a very sensitive indicator of mental health issues [38]. In the SAMINOR 1 study of Bakken et al. [39], with participants mainly from same areas as in the SAMINOR 2 study, the prevalence of insomnia and use of hypnotics in the core Sami group was only half of that in the non-Sami. In the Sami culture, living in accordance with nature is highly valued. Their long history of adaptation to natural processes such as light and weather may have increased their tolerance for accompanying mental stress responses and insomnia symptoms causing less need for alleviation.

The cross-cultural validity of the HSCL-10 in our study is strengthened by finding nearly identical prevalence rates of mental stress across the compared cultural groups, following adjustment for education and income. However, this finding contrasts international studies showing substantially lower rates of common mental disorders in Eastern Asia as compared to English-speaking counties of the West [20,21]. These studies have mainly used Western culturally developed instruments such as the Composite International Diagnostic Instrument (CIDI), and it has been recommended that culture-specific expressions of mental distress should be included when studying mental disorders across ethnic diverse groups [18]. Consequently, we cannot exclude the existence of culture-specific Sami expressions of mental stress not captured by HSCL 10.

### Limitation and strengths of the current study

The strengths of the study were its epidemiological design, the broad coverage of communities included and the rigorous measurement of ethnicity. Despite the large sample size and extremely high statistical power, the low participation rate (~27%) may have biased the results. Since Sami ethnicity is not included in official Norwegian registers, it was not possible to estimate potential differences in characteristics of the ethnic subgroups of the study population as compared with corresponding subgroups in the general population. However, a previous comparison between participants in the SAMINOR 1 and 2 studies showed that despite a considerably higher participation rate in SAMINOR 1 (60.9%), the proportion of participants classified as Sami did not differ between the 2 studies [40]. We may therefore assume that the proportion of non-respondents in SAMINOR 2 is comparably distributed among the Sami and the non-Sami.

The declining interest to participate in health surveys during the last decades is a general problem in epidemiological research. One may also suspect that individuals who are more familiar with a traditional narrative approach will be less inclined to respond to a Western questionnaire approach such as the SAMINOR 2 study. However, the degree of biases was most likely low for the CFA invariance analyses, the most important analyses in the current study, which examined interrelationships between variables rather than means or prevalence estimates.

## Conclusion

We conclude that the items included in the HSCL-10 are largely interpreted in a similar way by the ethnic sub-groups included in our study. This indicates that this instrument is applicable in Norwegian Sami populations with the same cut-off criteria of 1.85 as indicative of mental disorders. The confirmatory factor analysis also showed mediocre fit for the HSCL-10 as a single dimension. A 2-factor model accounting for the 6 depression and 4 anxiety items separately did not improve fit noticeably, whereas an abbreviated HSCL version solely accounting for the 6 depression items fitted the data well. These items may therefore be used to target depression symptomatology more specifically and valid as compared to the HSCL-10. However, one should still conduct qualitative studies looking for culture-specific expressions of mental stress.
